# Preparation of a Highly Porous Carbon Material Based on Quinoa Husk and Its Application for Removal of Dyes by Adsorption

**DOI:** 10.3390/ma11081407

**Published:** 2018-08-11

**Authors:** Siji Chen, Shanshan Tang, Yang Sun, Gang Wang, Huan Chen, Xiaoxiao Yu, Yingjie Su, Guang Chen

**Affiliations:** College of Life Sciences, Jilin Agricultural University, Changchun 130118, China; 18638342679@163.com (S.C.); tangshanshan81@163.com (S.T.); 15843052121@163.com (Y.Sun.); wanggang@jlau.edu.cn (G.W.); chjlau@163.com (H.C.); yx8751@sina.com (X.Y.); suyj0923@163.com (Y.Su.)

**Keywords:** quinoa husk, porous carbon, adsorption, malachite green, water treatment

## Abstract

A porous carbon material was prepared from quinoa husk (QH) by carbonization and chemical activation with KOH. A series of experiments, including SEM (Scanning electron microscopy), FT-IR (Fourier transform infrared), XRD (X-ray diffraction), Raman, X-ray photoelectron spectroscopy (XPS), and N_2_ adsorption/desorption, were carried out on the porous carbon produced from quinoa husk (PC–QH). The results showed that PC–QH was mainly composed of activated carbon and graphite. Moreover, PC–QH exhibited a high level of porosity with a BET (the Brunauer–Emmett–Teller theory) surface area of 1713 m^2^ g^−1^. As a representative dye, malachite green (MG) was selected to evaluate the performance of PC–QH to absorb the contaminants in dyeing wastewater. In batch adsorption experiments, PC–QH exhibited a high adsorption rate toward malachite green (MG). An uptake capacity of 599.90 mg g^−1^ was achieved in the initial 5 min, and the MG adsorption capacity of PC–QH reached 1365.10 mg g^−1^, which was higher than many other adsorbents. The adsorption data were well fitted with the Freundlich isotherm model and the pseudo-second-order kinetic model. PC–QH also displayed a high absorption rate to rhodamine B (RhB), methyl violet (MV), methylene blue (MB), and methyl orange (MO). The results in this study suggest that PC–QH can be a promising adsorbent for quick treatment of dyeing wastewater.

## 1. Introduction

As a high performance material, porous carbon has been widely used in the fields of catalysis, adsorption, separation, gas storage, energy storage, electrochemistry, etc., due to its unique advantages such as high specific surface area, large pore volume, special functional groups, and excellent chemical stability [1,2,3]. In recent years, studies on porous carbon derived from biomass have attracted much interest [4,5]. Biomass materials, like agricultural wastes, are inexpensive, abundant, and readily available. Once they are discarded or burned inappropriately, the ecological environment would be seriously polluted. However, these materials can also be utilized as sources of the production of porous carbon materials. In fact, carbon materials derived from biomass have already been used in the fields of supercapacitors [6,7], catalysis [8], electrocatalysis [9], adsorption of dyes and heavy metal ions [10], and so on.

The treatment of dyeing wastewater is a major application for porous carbon materials. Dyes are common contaminants present in wastewater. Due to their toxicity and carcinogenicity, it is imperative to remove the dyes from the wastewater. Among available technologies, adsorption has been regarded as the most promising approach because of its cost-effectiveness and feasibility. Over the past few decades, many biomass materials have been employed to prepare porous carbon materials with high adsorption capacities for dyes [11,12]. As a result, the dye adsorption has become an effective and reliable method for evaluating the performance of porous carbon materials.

Quinoa (*Chenopodium quinoa* Willd), a plant in Chenopodiceae family, is native to the Andean Region, South America. Nowadays, quinoa has been cultivated worldwide, owing to the high nutritional value of the seeds and its extraordinary adaptability to various agroecological conditions [13,14]. After the edible seeds are taken out, quinoa husk (QH), which is produced in large quantities, should be cautiously treated as an agricultural waste material. Any inappropriate treatment of QH may lead to potential environmental problems. QH mainly consists of cellulose, hemicellulose, and lignin. From our point of view, it can become an inexpensive and available source for the production of highly porous and high-performance carbon materials. So far, however, few reports are available on the preparation and application of porous carbon materials derived from QH (PC–QH).

Herein, QH was first carbonized and then chemically activated with KOH, with the aim to prepare porous carbon materials by using agricultural wastes and thus alleviate the environmental stresses caused by agricultural production. The performances of PC–QH were evaluated through the removal of dyes from aqueous solutions. The adsorption thermodynamics and kinetics of malachite green (MG) onto PC–QH were investigated. Moreover, the adsorption capacities and selectivity of PC–QH for dyes were evaluated with rhodamine B (RhB), methyl violet (MV), methylene blue (MB), and methyl orange (MO) as the model dyes.

## 2. Materials and Methods

### 2.1. Chemical and Materials

All chemicals and reagents were of analytical reagent grade and provided by Beijing chemical plant (Beijing, China). All of the dyes were purchased from Aladdin Chemical (Shanghai) Co., Ltd. (Shanghai, China). Deionized water was used in all preparation and flushing processes. 

### 2.2. Preparation of PC–QH

The quinoa used in this study was planted in the experiment plots of Jilin Agricultural University located in Changchun, northeast of China. QH, an agricultural solid waste, was used as the precursor for the preparation of powdered activated carbon. In a typical process, QH was washed with distilled water to remove the dusts and water-soluble impurities, and then dried at 80 °C for 12 h. The dried QH was carbonized in a tube furnace at a temperature of 500–650 °C for 90 min with a heating rate of 5 °C min^−1^ under N_2_ flow. No obvious difference was observed between the results under these temperatures by powder X-ray diffraction (XRD) test (Figure S1, Supplementary Materials). Thus, 500 °C was chosen for the carbonization of QH to save energy. The furnace was cooled to room temperature. The as-prepared carbon sample was mixed with KOH at a weight ratio of 1:3. Under N_2_ flow, the mixture was activated at 650 °C for 90 min with the heating rate of 5 °C min^−1^. The activated product was washed with HCl (0.1 mol L^−1^) and water until a neutral pH value of about 7 was reached. Finally, PC–QH was obtained after being dried at 80 °C for 12 h.

### 2.3. Batch Equilibrium Studies

MG, RhB, MV, MB, and MO (Figure S2, Supplementary Materials) were used as dye models to evaluate the adsorption capacities of samples. Dyes of commercial purity were used without further purification. All adsorption tests were performed under ambient conditions (298 ± 2 K) in the aqueous solutions. The pH values (3, 5, 7, 9, and 11) of solutions were adjusted using HCl or NaOH solutions. The initial and equilibrium concentrations were measured utilizing a UV-Vis (Agilent, Palo Alto, CA, USA) spectrophotometer at the wavelengths of 618, 554, 579, 664, and 463 nm for MG, RhB, MV, MB, and MO, respectively.

MG was selected for the adsorption isotherm and kinetic studies. In a typical process, the samples of QH-based carbon (10 mg) were added into the MG solutions (100 mL). The suspension would be shaken in dark at 150 rpm. At predetermined time intervals, 2 mL of the suspension was taken, centrifuged, and analyzed by UV-Vis absorption spectroscopy at the maximum wavelength of MG (618 nm).

The amount of adsorbed dye at equilibrium, *q_e_* (mg g^−1^), was calculated using the following Equation:

(1) $$q_{e}=\frac{\left(C_{0}-C_{e}\right)\times V}{W}$$

where *C_o_* and *C_e_* (mg L^−1^) are the initial and equilibrium concentrations of the dye, respectively. *V* (L) is the volume of dye solution. *W* (g) is the mass of dried samples.

### 2.4. Kinetic and Equilibrium Models

The adsorption kinetics of MG on carbon samples were studied using three common models: pseudo-first-order, pseudo-second-order, and intraparticle-diffusion models:

Pseudo-first-order Equation:

(2) $$\mathrm{lg}\left(q_{e}-q_{t}\right)=\mathrm{lg}q_{e}-\frac{K_{1}}{2.303}t$$

where *K*_1_ (min^−1^) is the pseudo-first-order rate constant, *q_t_* and *q_e_* (mg g^−1^) are the adsorption capacities at time *t* (min) and equilibrium, respectively.

Pseudo-second-order kinetic Equation:

(3) $$\frac{t}{q_{e}}=\frac{1}{K_{2}q_{e}^{2}}+\frac{t}{q_{e}}$$

where *K*_2_ (g mg^−1^ min^−1^) is the pseudo-second-order rate constant.

Intraparticle-diffusion Equation:

(4) $$q_{t}=K_{i}t^{0.5}$$

where *K_i_* (mg g^−1^ min^−0.5^) is the intraparticle diffusion rate constant. 

Two adsorption isotherm models were used to investigate the interactions between adsorbates and adsorbents:

The Langmuir isotherm Equation:

(5) $$\frac{C_{e}}{q_{e}}=\frac{C_{e}}{q_{m}}+\frac{1}{K_{L}q_{m}}$$

where *K_L_* (L mg^−1^) is Langmuir constant, *q_m_* is the maximum adsorption capacity.

Freundlich Equation:

(6) $$\mathrm{lg}q_{e}=\mathrm{lg}K_{F}+\frac{1}{n}\mathrm{lg}C_{e}$$

where *K_F_* and *n* are Freundlich constant and heterogeneity factor, respectively.

### 2.5. Reusability of PC–QH

The stability and reusability of PC–QH for removing MG were investigated through multicycle tests. In a typical process, the sample of PC–QH (30 mg) was added into the MG solutions (100 mL, 200 mg g^−1^). After each cycle, the used PC–QH would be separated from the solution by filtration and then activated with the solutions of NaCl and DMF under ultrasound at room temperature to remove the adsorbed MG. The re-generated PC–QH powder would be dried at 80 °C for 12 h and reused as absorbents in future.

### 2.6. Characterization

Scanning electron microscopy (SEM) experiments were performed on a SHIMADZU SSX-550 microscope (Shimadzu, Kyoto, Japan). Fourier transform infrared (FT-IR) patterns were obtained using a Bruker IFS 66V/S spectrometer (Bruker, Hamburg, Germany) with the KBr tablet method at a resolution of 1 cm^−1^ between 400 and 6000 cm^−1^. XRD pattern was measured using a Bruker D8 (Bruker, Hamburg, Germany) diffractometer with Cu-Kα X-ray source. The specific surface area calculated on the basis of the Brunauer–Emmett–Teller (BET) theory and the pore size distribution analyzed with the non-local density functional theory (NLDFT) were measured using N_2_ adsorption–desorption measurements (ASAP 2020, Micromeritics, Norcross, GA, USA). The samples were degassed at 180 °C for 12 h under vacuum before the measurement, and the data were recorded at liquid nitrogen temperature. The surface chemistry was analyzed by X-ray photoelectron spectroscopy (XPS, Escalab 250, Thermo Fisher, Waltham, MA, USA) equipped with Mg-Kα X-ray source. Raman study of the carbon material was carried out using an inVia Raman spectrometer (Renishaw, Gloucestershire, UK) with the laser wavelength of 488 nm. The concentrations of the dye solutions were determined using an Agilent Cary 300 UV-Vis spectrophotometer (Agilent, Palo Alto, CA, USA). 

## 3. Results

### 3.1. Characterization of PC–QH

**Figure 1:**
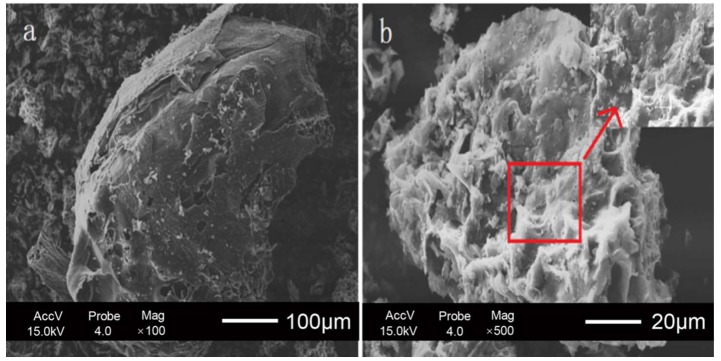
SEM images of QH-based carbon materials (**a**) before activation and (**b**) after activation with KOH.

The SEM images of the QH-based carbon materials before activation (C–QH) and after activation (PC–QH) are shown in Figure 1. From Figure 1a, we can see some macropores in the surface of C–QH derived from the natural structures of QH. After activation, abundant pores can be observed, which may result from the reaction of C–QH and KOH. The results indicate that KOH can serve as a reasonable and effective activator for treating carbon materials derived from biomass [2]. These pores may help increase the capacities of PC–QH to absorb dyes.

**Figure 2:**
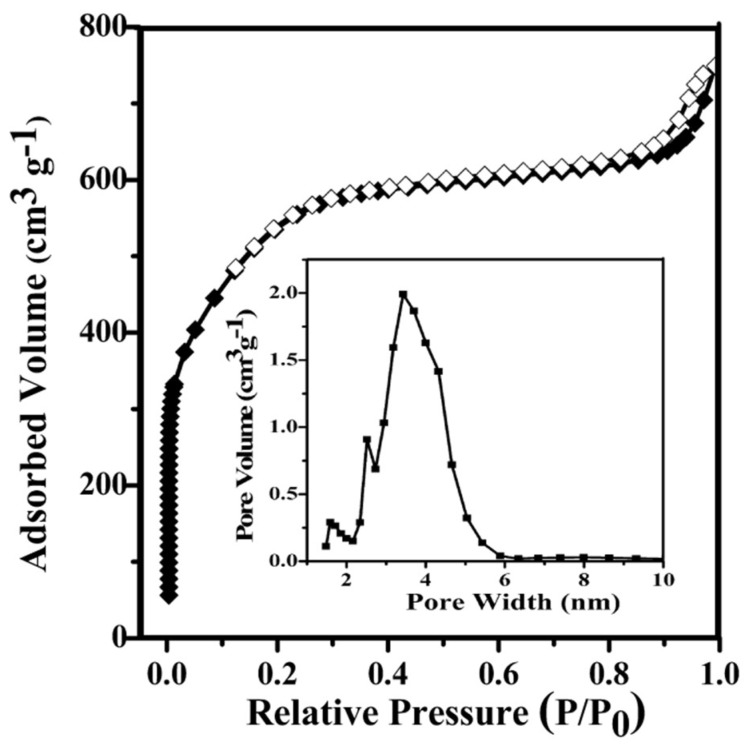
N_2_ adsorption–desorption isotherms and pore size distribution of PC–QH.

The FT-IR spectra of QH, C–QH, and PC–QH are shown in Figure S3 (Supplementary Materials). Cellulose, hemicellulose, and lignin are main components of QH. In the IR pattern of QH, the bands and peaks assigned to the groups of these components may be observed [15,16,17,18,19,20]. The bands at 3400 cm^−1^ and 2900 cm^−1^ can be assigned to O–H stretching of hydroxyl groups or adsorbed water [15,16]. The band appearing at 1393 cm^−1^ and the bands at 2893 cm^−1^ and 1312 cm^−1^ can be attributed to C–H stretching or deformation from CH_2_ to CH_3_ [16,17,18]. The bands at 1627 cm^−1^ and 1237 cm^−1^ are assigned to C=O and C–O–C, respectively [16,17]. In addition, the peak position appearing at 1096 cm^−1^ can be assigned to ring vibration in a large aromatic skeleton, which is generally found in the carbonaceous materials [17]. In the IR patterns of C–QH and PC–QH, the bands at 3400, 1393, and 1096 cm^−1^ may be observed, and many bands disappeared in the pattern of QH, indicating that QH has been successfully carbonized [21]. N_2_ adsorption experiments were carried out to characterize the porous structure of produced carbon materials. Table 1 shows the N_2_ adsorption data for C–QH and PC–QH. It can be seen that the BET surface area of C–QH is 0.42 m^2^ g^−1^. This indicates that C–QH is not a porous material. However, the specific surface area of PC–QH increased after being treated under different activation conditions, indicating the effectiveness of KOH to activate QH. The specific surface area of PC–QH varied when treated under different activation conditions. At the same activation time and alkali carbon ratio, the BET surface areas of PC–QH activated at 600 °C (760 m^2^ g^−1^) and 700 °C (120 m^2^ g^−1^) were both lower than that at 650 °C (1713 m^2^ g^−1^). At the same alkali carbon ratio and temperature, the BET surface areas of PC–QH activated at 60 min (228 m^2^ g^−1^) and 120 min (174 m^2^ g^−1^) were both lower than that at 90 min (1713 m^2^ g^−1^). At the same temperature and time, the BET surface areas of PC–QH activated at the ratio of alkali to carbon 2:1 (634 m^2^ g^−1^) and 4:1 (437 m^2^ g^−1^) were both lower than that at 3:1 (1713 m^2^ g^−1^). However, after being treated with KOH as the ratio of alkali to carbon 3:1 at 650 °C for 90 min, the BET surface area of PC–QH increased to 1713 m^2^ g^−1^. Thus, it can be concluded that the abundant pores were formed during the process of activation rather than during the process of carbonization. This is in agreement with the conclusion drawn from the SEM surface analysis (Figure 1). These results revealed that the PC–QH could be regarded as a highly porous carbon material. As shown in Figure 2, the rapid adsorption progress occurs at P/P_0_ = 0~0.3, indicating that there are micropores present in PC–QH. At P/P_0_ = 0.4~1.0, PC–QH shows the typical IV isotherm with a clear H3-type hysteresis loop, implying that there are a large number of mesopores in PC–QH [22]. These results can also be seen from the pore width distribution chart.

**Figure 3:**
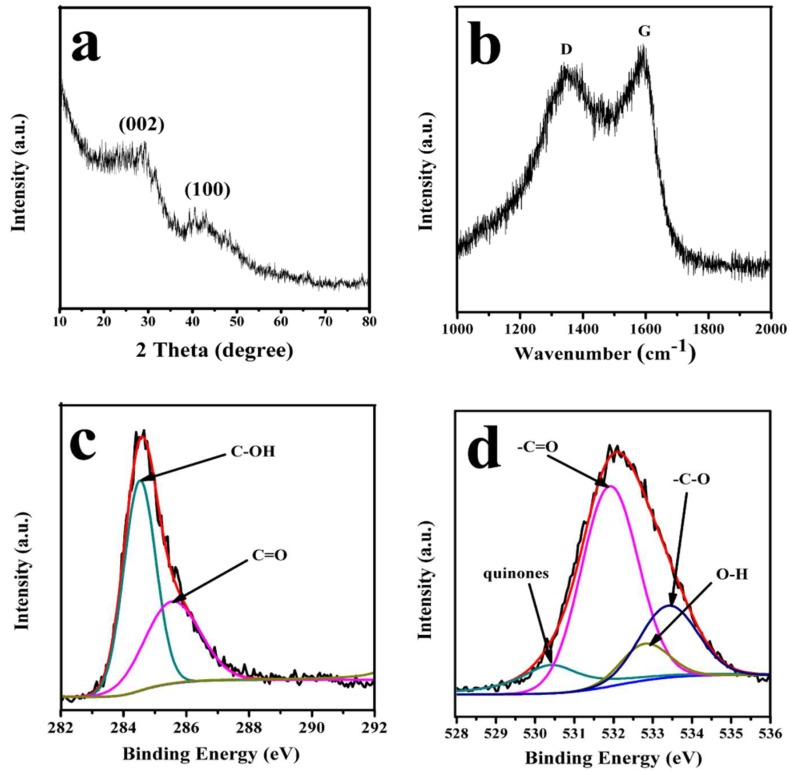
(**a**) XRD pattern and (**b**) Raman spectrum of PC–QH, (**c**) C1s peaks of PC–QH and (**d**) O1s peaks of PC–QH.

The crystal structure, elemental composition, and surface chemistry of PC–QH were studied by XRD (Figure 3a), Raman (Figure 3b), and XPS (Figure 3c,d), respectively. As shown in Figure 3a, in the XRD pattern, PC–QH exhibited two diffraction peaks within the range of 20–50°. This may be assigned to the (002) and (100) planes of graphite structure [18]. The intensities of both two peaks were weak, and both two peaks were broad peaks. The reason might be that PC–QH contains amorphous carbon [23]. This is in agreement with the result of Raman spectrum. As shown in Figure 3b, the D peak at around 1330 cm^−1^ represents the amorphous carbon with smaller grain size, and the G peak at around 1590 cm^−1^ represents the crystalline graphite [24]. Thus, it can be concluded that PC–QH is mainly composed of amorphous carbon and graphite. The surface chemical properties of PC–QH were studied by the XPS (Figure 3c,d). Figure 3c shows that the C1s high resolution spectrum of PC–QH can be fitted into three separate peak positions, which are C–OH (284.6 eV) and C=O (285.9 eV), respectively [25,26]. As shown in Figure 3d, the O1s high resolution spectrum of PC–QH can be fitted into four separate peak positions, which are quinones (530.4 eV), C=O (531.9 eV), C–O (532.8 eV), and –OH (533.4 eV), respectively [21,25,26,27]. 

### 3.2. Preparation of Activated Carbon

#### 3.2.1. Effect of Activation Temperature

Usually, the activation temperature has a great impact on the specific surface area of the prepared materials [28].

**Figure 4:**
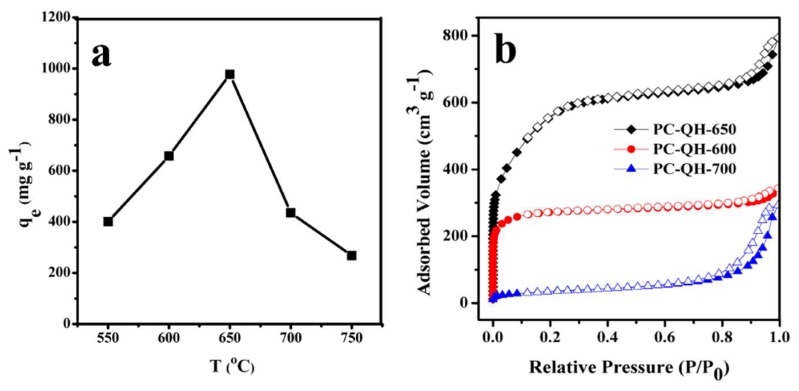
Effect of activation temperature on (**a**) the adsorption capacities of PC–QH to MG (adsorbent: 0.1 g L^−1^; *C*_0_: 200 mg L^−1^; *V*: 100 mL; pH: 4.6 ± 0.2) and (**b**) N_2_ adsorption–desorption isotherms.

Figure 4a shows the effect of activation temperature on the capacity of PC–QH to absorb MG. The alkali/carbon ratio is 3:1, and the activation time is 90 min. It can be seen from Figure 4a that as the activation temperature increased from 500 °C to 650 °C, the adsorption capacity of PC–QH to MG increased, with the maximum adsorption capacity being 977.43 mg g^−1^. It might be explained that, when the activation temperature increased, the activation rate increased and a large number of pore structures were formed, which could increase the adsorption capacity of PC–QH to MG. However, as the activation temperature increased further, the adsorption capacity of PC–QH to MG decreased, exerting a negative impact on the adsorption of dyes [29]. The above analysis is in agreement with the results from N_2_ adsorption experiments. Figure 4b shows the N_2_ adsorption–desorption isotherms of PC–QH at different temperatures. PC–QH-650 means that the activation temperature is 650 °C, PC–QH-600 means that the activation temperature is 600 °C, and PC–QH-700 means that the activation temperature is 700 °C. When the temperature increased from 600 °C to 650 °C, the BET surface area of samples increased from 760 m^2^ g^−1^ to 1713 m^2^ g^−1^, which might be attributed to the increase in not only the number of pores but also the volume of pores. When the temperature further increased to 700 °C, the BET surface area decreased to 120 m^2^ g^−1^, and the average pore size increased from 2.87 nm to 15.01 nm (Table 1), indicating that C–QH was overactivated. As a result, the most appropriate activation temperature should be 650 °C.

#### 3.2.2. Effect of Activation Time

**Figure 5:**
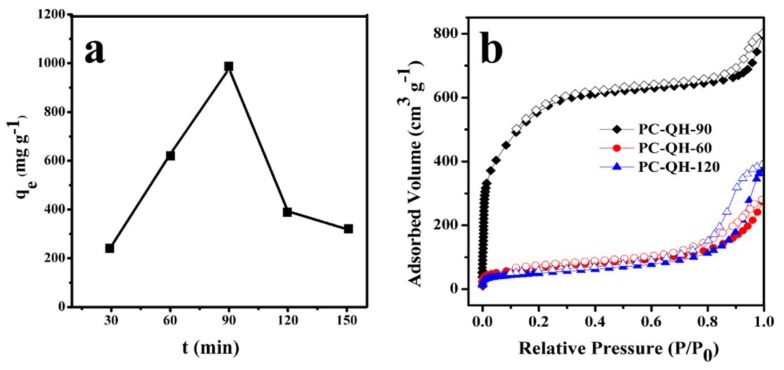
Effect of activation time on (**a**) the adsorption capacities of PC–QH to MG (adsorbent: 0.1 g L^−1^; *C*_0_: 200 mg L^−1^; *V*: 100 mL; pH: 4.6 ± 0.2) and (**b**) N_2_ adsorption–desorption isotherms.

Figure 5a shows the effect of activation time on the adsorption capacity of PC–QH to MG. The alkali/carbon ratio is 3:1, and the activation temperature is 650 °C. As shown in Figure 5a, the adsorption capacity of PC–QH to MG gradually increased with the increment of activation time. When the activation time reached 90 min, the adsorption capacity of PC–QH to MG reached the highest level, which was 977.43 mg g^−1^. However, when the activation time extended, the adsorption capacity of PC–QH to MG decreased. The reason might be that the micropores were enlarged and collapsed as time went on, and the rate of micropores destruction finally exceeded the rate of new pores formation [30]. Thus, the adsorption capacity of PC–QH to MG decreased. Figure 5b shows the N_2_ adsorption–desorption isotherms of PC–QH at different activation times. In Figure 5b, PC–QH-60, PC–QH-90, and PC–QH-120 mean that the activation time is 60 min, 90 min, and 120 min, respectively. The BET surface areas of PC–QH-60 and PC–QH-120 were 228 and 174 m^2^ g^−1^ (Table 1), respectively, which were smaller than that of PC–QH-90 (1713 m^2^ g^−1^, Table 1). Thus, the optimum activation time was determined to be 90 min.

#### 3.2.3. Effect of Alkali/Carbon Ratio

In this study, KOH was used as the activator, since the hydroxides (specifically KOH) are frequently used as activators for the production of activated carbons [31].

**Figure 6:**
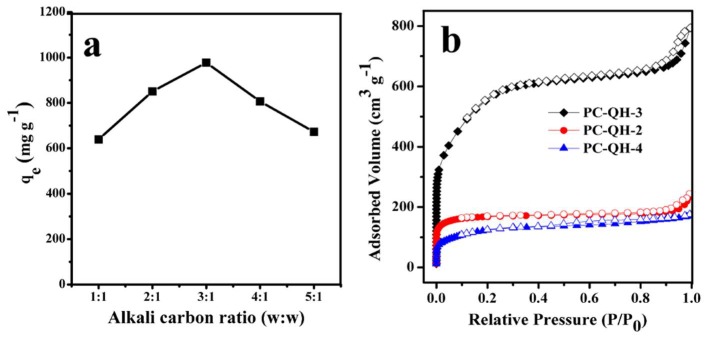
Effect of alkali/carbon ratio on (**a**) the adsorption capacity of PC–QH to MG (adsorbent: 0.1 g L^−1^; *C*_0_: 200 mg L^−1^; *V*: 100 mL; pH: 4.6 ± 0.2) and (**b**) N_2_ adsorption–desorption isotherms.

Figure 6a shows the effect of alkali/carbon ratio on the adsorption capacity of PC–QH to MG. The activation time was 90 min, and the activation temperature was 650 °C. According to Figure 6a, we can find that with the increase in alkali/carbon ratio, the adsorption capacity of PC–QH to MG first increased and then decreased. The optimal alkali/carbon ratio is 3:1, which is consistent with the results of N_2_ adsorption experiments (Figure 6b). In Figure 6b, PC–QH-2, PC–QH-3, and PC–QH-4 mean that the alkali/carbon ratios were 2:1, 3:1, and 4:1, respectively. The BET surface areas of PC–QH-2 and PC–QH-4 were 634 and 437 m^2^ g^−1^ (Table 1), respectively, both of which were smaller than that of PC–QH-3 (1713 m^2^ g^−1^, Table 1). The results suggested when the alkali/carbon ratios were 1:1 and 2:1, carbon and activator reacted incompletely, and the pore structures were inadequately developed. However, the excess of alkali activator led to the decrease in BET surface area and porosity. The reason might be that the excessive reaction between the carbon and activator damaged the formed pores.

### 3.3. Adsorption Studies

#### 3.3.1. Effect of pH on Adsorption Capacity

**Figure 7:**
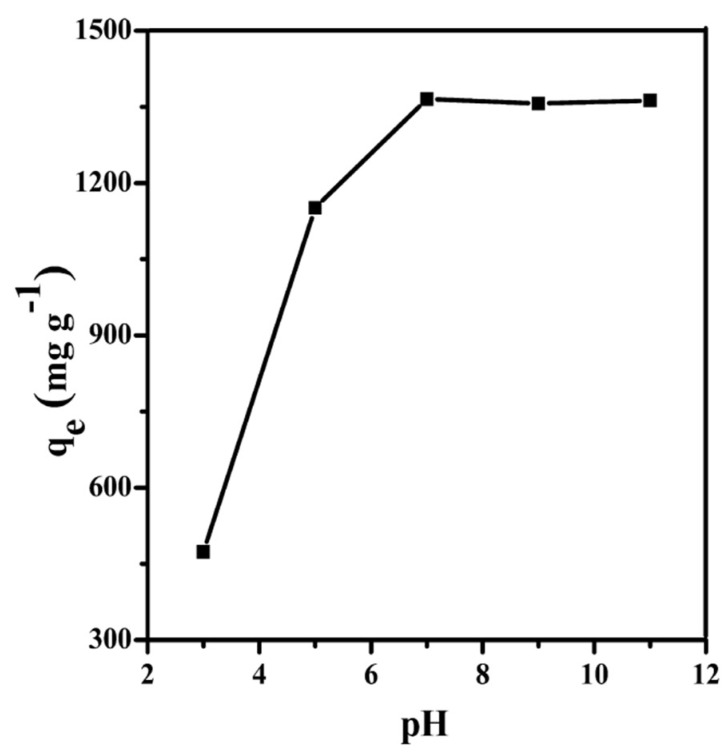
Effect of pH on adsorption capacity of PC–QH to MG (adsorbent: 0.1 g L^−1^; *C*_0_: 200 mg L^−1^; *V*: 100 mL).

Generally, the pH value of the solution may promote or suppress dye uptake by changing the surface of adsorbent and the chemical structure of dye [32,33]. Herein, we investigated the effect of the pH on the adsorption capacity of PC–QH to MG. As shown in Figure 7, as the pH values of solution increased, the adsorption capacity of PC–QH to MG increased markedly from 473.45 to 1365.10 mg g^−1^. Until the pH value of solution reached 7, the adsorption capacity of PC–QH to MG started to level off. Therefore, the optimum pH value with the highest adsorption capacity of PC–QH to MG was fixed to 7. The adsorption capacity of PC–QH to MG (1365.10 mg g^−1^) was higher than that of graphene and many other adsorbents prepared from biomass (Table S1, Supplementary Materials).

#### 3.3.2. Adsorption Kinetics

**Figure 8:**
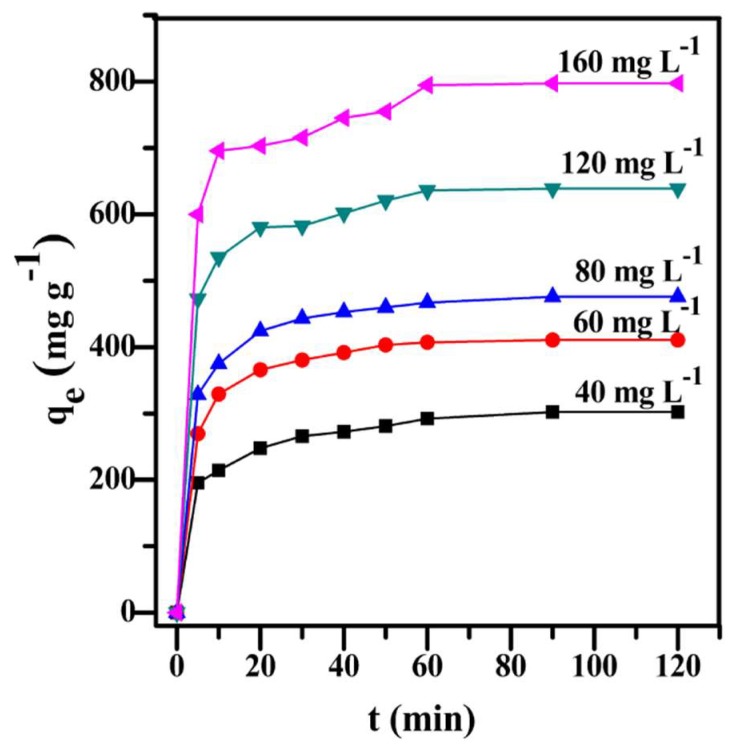
Effect of initial MG concentration on the adsorption ability of PC–QH (adsorbent: 0.1 g L^−1^; pH: 7; *V*: 100 mL).

The adsorption rate is an important parameter to evaluate the adsorption process of prepared adsorbents. The adsorption kinetics curves of PC–QH to MG with different initial concentrations are shown in Figure 8. The pseudo-first-order kinetic, pseudo-second-order kinetic, and Intra-particle diffusion models were used to estimate the rate constants, initial adsorption rates, and adsorption capacities of the prepared PC–QH. The fitting parameters are summarized in Table 2. As can be seen in Figure 8, the adsorption curves maintained the same trend. In the first 5 min, the adsorption capacity increased sharply and reached an equilibrium within 60 min. With the increase in the initial concentration of MG, the adsorption capacity of PC–QH to MG increased significantly. This revealed that higher concentration of dye solution could result in larger adsorption capacity of PC–QH to MG.

The Lagergren’s pseudo-first-order equation has been known as an earliest model to describe the adsorption rate on the basis of the adsorption capacity [34,35]. The kinetic parameters *K*_1_ and *q_e_* could be calculated from the intercept of the slope and lg(*q_e_* − *q_t_*) to *t*. The results showed that the correlation coefficient *R*^2^ was within the range of 0.7225–0.9864. In addition, the actual measured activated carbon adsorption values of PC–QH (302.12, 410.66, 475.84, 638.73, and 797.20) were higher than the theoretical adsorption values (129.51, 162.85, 167.19, 201.93, and 302.41). These results showed that the pseudo first-order kinetic model was not suitable for describing the adsorption process. 

The rate constant *K*_2_ and the maximum theoretical adsorption value of the pseudo second-order model can be determined from the intercept and slope; the intercept and slope can be obtained by calculating *t*/*q_t_* and *t*. In Table 2, a good linear relationship between *t*/*q_t_* and *t* can be seen that the correlation coefficient *R*^2^ was within the range of 0.9971 to 0.9998. In addition, the equilibrium adsorption value obtained from the experiment was consistent with the maximum theoretical adsorption of the calculated data. These results indicated that the adsorption of PC–QH to MG followed the pseudo-second-order kinetic model.

The results analyzed on the basis of the intra-particle diffusion model showed that the correlation coefficient *R*^2^ was within the range of 0.8759–0.9725, which was poorly fitted to the linear relationship of *q_t_* with respect to *t*^0.5^. The results showed that the adsorption process might not follow the intra-particle diffusion model.

#### 3.3.3. Adsorption Isotherms

**Figure 9:**
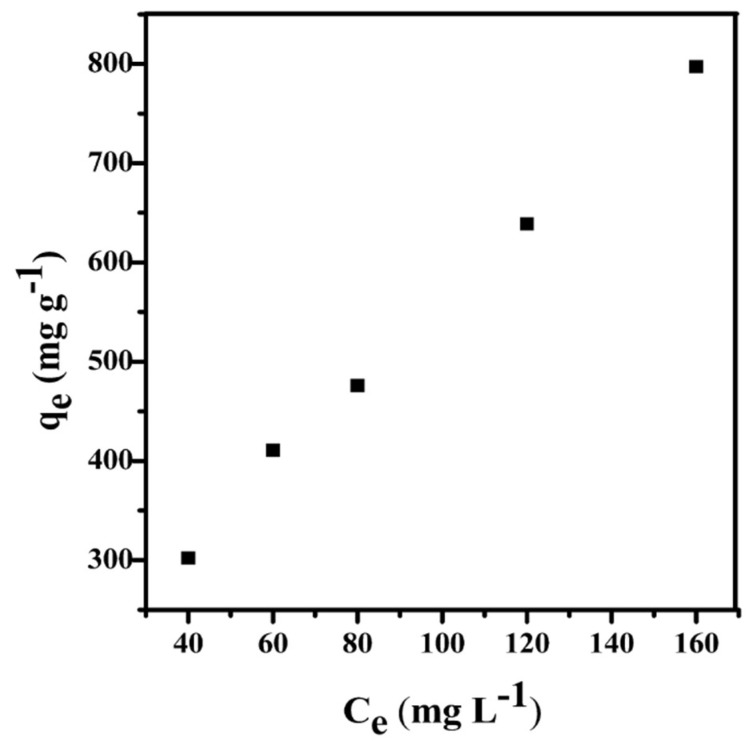
Adsorption isotherms of MG at 298 K.

Adsorption isotherms are commonly used to describe the interaction between adsorbates and carbonaceous adsorbents, and are thus critical to the optimization of adsorption mechanism pathways [36]. In order to describe the adsorption properties between the dye molecules and the adsorbent, the most commonly used isotherm models such as the Langmuir and Freundlich isotherm models were used to fit the experimental data. Figure 9 shows the adsorption isotherms of PC–QH to MG at 298 K. The corresponding data are shown in Table 3. The linear correlation coefficient *R*^2^ of adsorption Langmuir isotherm for PC–QH to MG was 0.9549 and the *R*^2^ of the Freundlich isotherm for PC–QH to MG was 0.9972. Therefore, the adsorption of PC–QH to MG is suitable for the Freundlich isotherm model.

### 3.4. Reusability of PC–QH

The stability and reusability of PC–QH for removing MG were investigated through multicycle tests. As shown in Figure S4 (Supplementary Materials), the adsorbent was still able to remove MG up to 90% albeit over three cycles, suggesting that PC–QH exhibits a good reusability.

### 3.5. Adsorption Selectivity of PC–QH

In some cases, adsorbents exhibit obviously different adsorption capacities for dyes with different charges, which may limit their applications in wastewater treatment. Herein, the adsorption performance of PC–QH to MB (cationic dye) and MO (anionic dye) were studied, due to their similar molecular structures and sizes [37]. The experiment results showed that the adsorption capacities of PC–QH to MB and MO reached 561.72 mg g^−1^ and 550.54 mg g^−1^ after 30 min, respectively. The results revealed that the adsorption of PC–QH to dye (cationic and anionic) did not show obvious ion selectivity. That is to say, PC–QH might be used widely to adsorb various dyes with different charges. Additionally, the quick adsorption capacities of PC–QH to RhB and MV reached 759.39 and 600.66 mg g^−1^, respectively. Thus, PC–QH can be considered as one of the most desirable adsorbents.

## 4. Conclusions

It is the first time that a porous carbon material was prepared using QH as the raw material. After the processes of carbonization and chemical activation, the produced carbon material, PC–QH, exhibited a large surface area and a high level of porosity. PC–QH exhibited a high adsorption rate toward MG with the adsorption capacity of 1365.10 mg g^−1^. The adsorption process of PC–QH to MG follows the pseudo-second-order kinetics and the Freundlich isotherm. PC–QH could be readily recycled and reused. Moreover, it could be widely used for adsorbing various dyes with different sizes and charges. The quick adsorption capacities of PC–QH to RhB, MV, MB, and MO reached 759.39, 600.66, 561.72, and 550.54 mg g^−1^, respectively. To sum up, this study could provide a foundation for subsequent studies on the preparation of carbon materials and investigation of the adsorption properties. In the future, we will further explore the preparation of highly porous carbon based on QH with different activation methods and applications in supercapacitors, electrocatalysis, adsorption of heavy metal ions, and so on.

## Figures and Tables

**Table 1:** The data of N_2_ adsorption for C–QH and PC–QH.

Samples	Activiation Conditions			S_BET_^2^ (m^2^ g^−1^)	V_micro_^3^ (cm^3^ g^−1^)	V_total_^4^ (cm^3^ g^−1^)	D_pore_^5^ (nm)
	T (°C)	t (min)	A/Cr ^1^				
C–QH	---	---	---	0.42	0.0001	0.0026	24.78
PC–QH	650	90	3:1	1713	0.53	1.23	2.87
PC–QH	600	90	3:1	760	0.34	0.53	2.79
PC–QH	700	90	3:1	120	0.01	0.45	15.01
PC–QH	650	60	3:1	228	0.05	0.42	2.44
PC–QH	650	120	3:1	174	0.01	0.58	13.38
PC–QH	650	90	2:1	634	0.25	0.34	2.14
PC–QH	650	90	4:1	437	0.14	0.27	2.43

^1^ A/Cr is alkali/carbon ratio, ^2^ S_BET_ (m^2^ g^−1^) is the BET surface area, ^3^ V_micro_ (cm^3^ g^−1^) is the volume of micropores, ^4^ V_total_ (cm^3^ g^−1^) is the total pore volume, and ^5^ D_pore_ (nm) is the average pore diameter.

**Table 2:** Kinetic models parameters of PC–QH to MG.

*C*_0_ (mg L^−1^)	*q_e_* (mg g^−1^)	Pseudo-First-Order Kinetic			Pseudo-Second-Order Kinetic			Intra-Particle Kinetic		
		*K*_1_ (min^−1^)	*Q_e.cat_* (mg g^−1^)	*R* ^2^	*K*_2_ (g mg^−1^ min^−1^)	*Q_e.cat_* (mg g^−1^)	*R* ^2^	*K*_3_ (mg g^−1^ min^−0.5^)	*Q_e.cat_* (mg g^−1^)	*R* ^2^
40	302.12	0.0398	129.51	0.9785	0.0008	303.03	0.9982	17.397	161.96	0.9725
60	410.66	0.0633	162.85	0.9864	0.0007	416.67	0.9998	22.733	245.10	0.8884
80	475.84	0.0405	167.19	0.9821	0.0008	500.01	0.9998	24.529	294.47	0.9156
120	638.73	0.0240	201.93	0.9512	0.0006	666.67	0.9987	26.375	438.08	0.9220
160	797.20	0.0580	302.41	0.7225	0.0005	833.33	0.9971	28.260	568.27	0.8759

**Table 3:** Isotherms parameters of PC–QH to MG.

Langmuir			Freundlich		
*Q_m_* (mg g^−1^)	*K_L_* (L mg^−1^)	*R* ^2^	*K_F_* (mg g^−1^ (L mg^−1^)^1/*n*^)	*n*	*R* ^2^
1666.67	0.0053	0.9549	23.9332	1.4533	0.9972

## Supplementary Materials

The following are available online at http://www.mdpi.com/1996-1944/11/8/1407/s1, Figure S1: The structures of malachite green (MG), rhodamine B (RhB), methyl violet (MV), methylene blue (MB), and methyl orange (MO), Figure S2: The reusability of PC–QH (pH: 7; *C*_0_: 100 mg g^−1^; *V*: 100 mL), Figure S3: FT-IR spectra of QH, C-QH, and PC-QH, Figure S4: The reusability of PC-QH (pH: 7; *C_0_*: 100 mg g^−1^; *V*: 100 mL), Table S1: Comparison of the adsorption capacity of PC-QH to MG with other adsorbents.
