# Supplementary material for: Preparation of a Highly Porous Carbon Material Based on Quinoa Husk and Its Application for Removal of Dyes by Adsorption
Source: Materials (Basel). 2018 Aug 11;11(8):1407. doi: 10.3390/ma11081407 (PMC6119990; doi:10.3390/ma11081407)
Supplement: Supplementary file 1 [file materials-11-01407-s001.pdf]

Preparation of a Highly Porous Carbon Material Based on Quinoa Husk and Its Application for Removal of Dyes BY Adsorption

Siji Chen, Shanshan Tang, Yang Sun, Gang Wang, Huan Chen, Xiaoxiao Yu, Yingjie Su, Guang Chen*

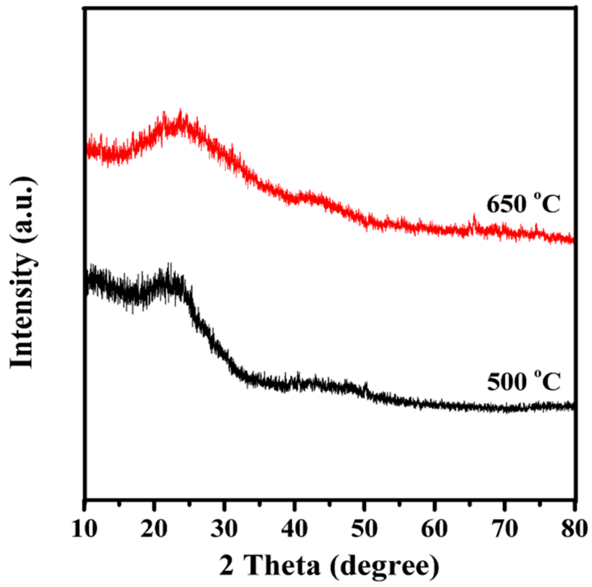

Figure S1. XRD of C-QH at different carbonization temperatures.

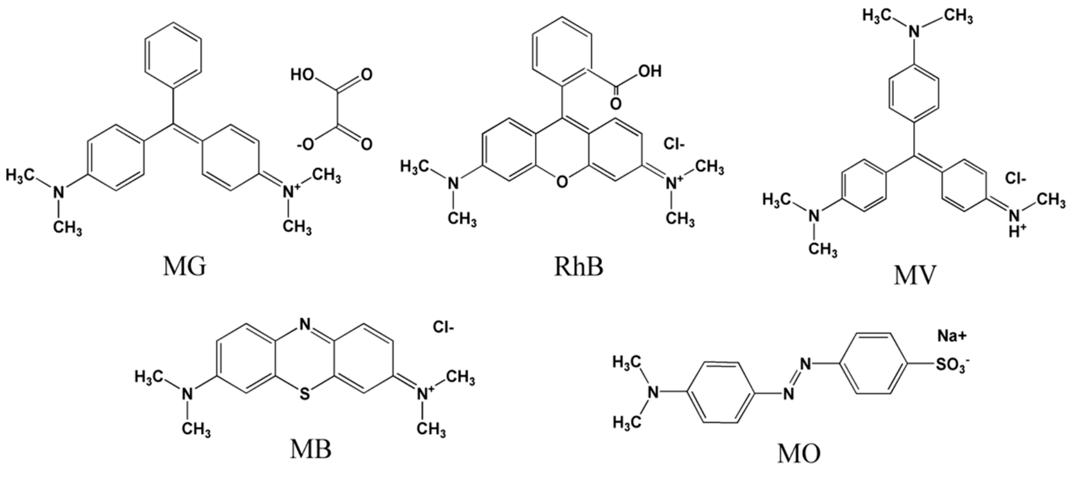

Figure S2. The structures of malachite green (MG), rhodamine B (RhB), methyl violet (MV), methylene blue (MB), and methyl orange (MO).

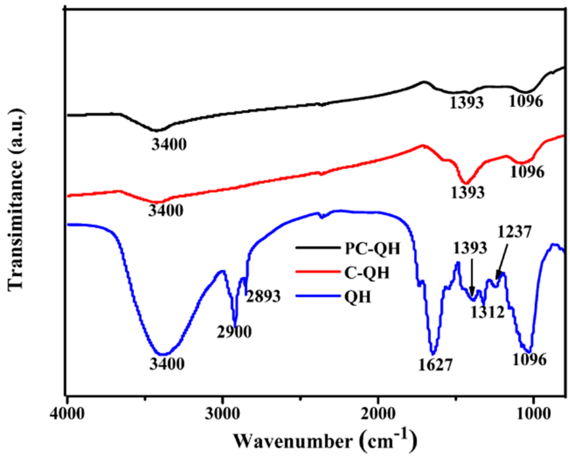

Figure S3. FT-IR spectra of QH, C-QH, and PC-QH.

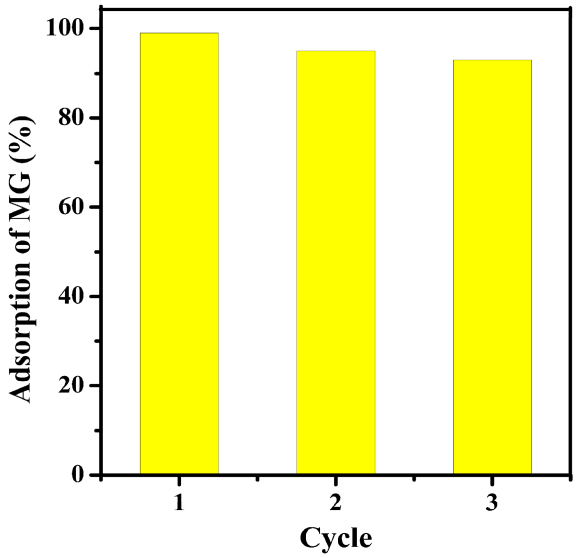

Figure S4. The reusability of PC-QH (pH: 7; C_0: 100 mg g-1; V: 100 mL).

Table S1. Comparison of the adsorption capacity of PC-QH to MG with other adsorbents.

Dye	Adsorbents	q_m (mg g^{-1})	Refs.
MG	Cellulose	2.42	[S1]
	Hen feathers	2.82	[S2]
	Neem sawdust	4.35	[S3]
	Sugarcane dust	4.88	[S4]
	Novel chitosan ionic liquid beads	8.07	[S5]
	Arundo donax root carbon	8.69	[S6]
	Ricinus communis based activated carbon	27.78	[S7]
	Carbonized pomegranate peel	31.45	[S8]
	Activated carbon prepared from Rubber wood sawdust	36.45	[S9]
	Borassus aethiopum flower biomass based activated carbon	48.48	[S10]
	Forestry waste mixture	52.61	[S11]
	Natural Carica papaya wood	52.63	[S12]
	Activated carbon derived from rice husks	57.14	[S13]
	Rattan sawdust	62.71	[S14]
	Rice husk bio-char	67.60	[S15]
	Rice husk	76.92	[S16]
	Adsorbent prepared from maize cob powder	80.64	[S17]
	Bivalve shell-treated Zea mays L. (maize) husk leaf	81.50	[S18]
	Beech sawdust	83.21	[S19]
	Walnut shell	90.80	[S20]
	Rice bran-based composites	93.68	[S21]
	Oak acorn peel	111.85	[S22]
	Modified sphagnum peat moss	121.95	[S23]
	Rice straw-derived char	148.74	[S24]
	Oil palm trunk fibre	149.35	[S25]
	Chitosan	166.00	[S26]
	Activated carbon prepared from almond gum	196.07	[S27]
	Jute fiber based carbon	196.08	[S28]
	Raw corn straw (RCS)	200.00	[S29]
	Ultrasound-assisted modified corn straw (MCS)	210.00	[S29]
	CO_2-activated porous carbon derived from cattail biomass	210.18	[S30]
	Cellulose nanofibril aerogels	212.70	[S31]
	Carbon aerogels	238.20	[S32]
	Activated carbon produced from spent tea leaves	256.40	[S33]
	Activated carbon prepared from bamboo	263.58	[S34]
	Covalently functionalized Fe_3O_4@SiO_2–graphene oxides core–shell	265.87	[S35]
	Fabrication of starch-graft-poly(acrylamide)/grapheneoxide/hydroxyapatite	297.00	[S36]
	Tetraethylenepentamine-functionalized Rosa canina-L fruits activated carbon	333.30	[S37]
	Biodegradable adsorbent based on amylopectin and poly	352.11	[S38]
	Cellulose modified with maleic	370.00	[S39]

Reduction of graphene oxide	416.70	[S40]
Reduced graphene oxide	476.20	[S41]
Magnetic graphene oxide	714.30	[S42]
TEMPO-oxidized cellulose beads	740.00	[S43]
Fibrous cellulose sulfate prepared from medical cotton waste	960.00	[S44]
Macroalgae-derived biochar (MDBC800)	5306.20	[S45]
PC-QH	1365.10	This work

References

S1 Sekhar, C.P.; Kalidhasan, S.; Rajesh, V.; Rajesh, N. Bio-polymer adsorbent for the removal of malachite green from aqueous solution. *Chemosphere* **2009**, 77, 842-847.

S2 Mittal, A. Adsorption kinetics of removal of a toxic dye, Malachite Green, from wastewater by using hen feathers. *J. Hazard. Mater.* **2006**, 113, 196-202.

S3 Khattri, S.D.; Singh, M.K. Removal of malachite green from dye wastewater using neem sawdust by adsorption. *J. Hazard. Mater.* **2009**, 167, 1089-1094.

S4 Khattri, S.D.; Singh, M.K. Colour Removal From Dye Wastewater Using Sugar Cane Dust as an Adsorbent. *Adsorpt. Sci. Technol.* **1999**, 17, 269–282.

S5 Naseeruteena, F.; Hamida, N.S.A.; Suaha, F.B.M.; Ngaha, W.S.W.; Mehamod, F.S. Adsorption of malachite green from aqueous solution by using novel chitosan ionic liquid beads. *Int. J. Biol. Macromol.* **2017**, 107, 1270-1277.

S6 Zhang, J.; Li, Y.; Zhang, C.; Jing, Y. Adsorption of malachite green from aqueous solution onto carbon prepared from Arundo donax root. *J. Hazard. Mater.* **2008**, 150, 774-782.

S7 Santhi, T.; Manonmani, S.; Smitha, T. Removal of malachite green from aqueous solution by activated carbon prepared from the epicarp of Ricinus communis by adsorption. *J. Hazard. Mater.* **2010**, 179, 178-186.

S8 Gündüz, F.; Bayrak, B. Biosorption of malachite green from an aqueous solution using pomegranate peel: Equilibrium modelling, kinetic and thermodynamic studies. *J. Mol. Liq.* **2017**, 243, 790-798.

S9 Kumar, K.V.; Sivanesan, S. Isotherms for Malachite Green onto rubber wood (Hevea brasiliensis) sawdust: Comparison of linear and non-linear methods. *Dyes Pigments.* **2007**, 72, 124-129.

S10 Nethaji, S.; Sivasamy, A.; Thennarasu, G. Saravanan, S. Adsorption of Malachite Green dye onto activated carbon derived from Borassus aethiopum flower biomass. *J. Hazard. Mater.* **2010**, 181, 271-280.

S11 Deniz, F.; Kepekci, R.A. Bioremoval of Malachite green from water sample by forestry waste mixture as potential biosorbent. *Microchem. J.* **2017**, 132, 172-178.

S12 Rangabhashiyam, S.; Sujata, L.; Balasubramanian, P. Biosorption characteristics of methylene blue and malachite green from simulated wastewater onto Carica papaya wood biosorbent. *Surfaces and Interfaces* **2018**, 10, 197-215.

S13 Rahman, I.A.; Saad, B.; Shaidan, S.; Sya Rizal, E.S. Adsorption characteristics of malachite green on activated carbon derived from rice husks produced by chemical-thermal process. *Bioresource Technol.* **2005**, 96, 1578-1583.

S14 Hameed, B.H.; El-Khaiary, M.I. Batch removal of malachite green from aqueous solutions by adsorption on oil palm trunk fibre: equilibrium isotherms and kinetic studies. *J. Hazard. Mater.* **2008**, 159, 574-579.

S15 Leng, L.; Yuan, X.; Zeng, G.; Shao, J.; Chen, X.; Wu, Z. Surface characterization of rice husk bio-char

produced by liquefaction and application for cationic dye (Malachite green) adsorption. *Fuel* **2015**, 155, 77-85.

S16 Rahman, I.A.; Saad, B.; Shaidan, S.; Rizal, E.S.S. Adsorption characteristics of malachite green on activated carbon derived from rice husks produced by chemical-thermal process. *Bioresource Technol.* **2005**, 96, 1578-1583.

S17 Sonawane, G.H.; Shrivastava, V.S. Kinetics of decolourization of malachite green from aqueous medium by maize cob (Zea maize): an agricultural solid waste. *Desalination* **2009**, 247, 430-441.

S18 Jalil, A.A.; Triwahyono, S.; Yaakob, M.R.; Azmi, Z.Z.A.; Sapawe, N.; Kamarudin, N.H.N. Utilization of bivalve shell-treated Zea mays L. (maize) husk leaf as a low-cost biosorbent for enhanced adsorption of malachite green. *Bioresource Technol.* **2012**, 120, 218-224.

S19 Witek-Krowiak, A. Analysis of influence of process conditions on kinetics of malachite green biosorption onto beech sawdust. *Chem. Eng. J.* **2011**, 171, 976-985.

S20 Dahri, M.K.; Kooh, M.R.R.; Lim, L.B.L. Water remediation using low cost adsorbent walnut shell for removal of malachite green: Equilibrium, kinetics, thermodynamic and regeneration studies. *J. Environ. Chem. Eng.* **2014**, 2, 1434-1444.

S21 Bhatti, H.N.; Jabeen, A.; Iqbal, M.; Noreen, S.; Naseem, Z. Adsorptive behavior of rice bran-based composites for malachite green dye: Isotherm, kinetic and thermodynamic studies. *J. Mol. Liq.* **2017**, 237, 322-333.

S22 Kuppusamy, S.; Venkateswarlu, K.; Thavamani, P.; Bok Lee, Y.; Naidu, R.; Megharaj, M. Quercus robur acorn peel as a novel coagulating adsorbent for cationic dye removal from aquatic ecosystems. *Ecol. Eng.* **2017**, 101, 3-8.

S23 Hemmati, F.; Norouzbeigi, R.; Sarbisheh, F.; Shayesteh, H. Malachite green removal using modified sphagnum peat moss as a low-cost biosorbent: Kinetic, equilibrium and thermodynamic studies. *J. Taiwan Inst. Chem. E.* **2015**, 58, 482-489.

S24 Hameed, B.H.; El-Khaiary, M.I. Kinetics and equilibrium studies of malachite green adsorption on rice straw-derived char. *J. Hazard. Mater.* **2008**, 153, 701-708.

S25 Hameed, B.H.; El-Khaiary, M.I. Batch removal of malachite green from aqueous solutions by adsorption on oil palm trunk fibre: equilibrium isotherms and kinetic studies. *J. Hazard. Mater.* **2008**, 154, 237-244.

S26 Subramani, S.E.; Thinakaran, N. Isotherm, kinetic and thermodynamic studies on the adsorption behavior of textile dyes onto chitosan. *Process Saf. Environ.* **2016**, 106, 1-10.

S27 Bouaziz, F.; Koubaa, M.; Kallel, F.; Ghorbel, R.E.; Chaabouni, S.E. Adsorptive removal of malachite green from aqueous solutions by almond gum: Kinetic study and equilibrium isotherms. *Int. J. Biol. Macromol.* **2017**, 105, 56-65.

S28 Porkodi, K.; Kumar, K.V. Equilibrium, kinetics and mechanism modeling and simulation of basic and acid dyes sorption onto jute fiber carbon: Eosin yellow, malachite green and crystal violet single component systems. *J. Hazard. Mater.* **2007**, 143, 311–327.

S29 Lima, D.R.; Klein, L.; Dotto, G.L. Application of ultrasound modified corn straw as adsorbent for malachite green removal from synthetic and real effluents. *Environ. Sci. Pollut. Res.* **2017**, 24, 1-12.

S30 Yu, M.; Han, Y.; Li, J.; Wang, L. CO_2-activated porous carbon derived from cattail biomass for removal of malachite green dye and application as supercapacitors. *Chem. Eng. J.* **2017**, 317, 493-502.

S31 Jiang, F.; Dinh, D.M.; Hsieh, Y.L. Adsorption and desorption of cationic malachite green dye on cellulose nanofibril aerogels. *Carbohyd Polym.* **2017**, 173, 286-294.

S32 Yu, M.; Han, Y.; Li, J.; Wang, L. Magnetic N-doped carbon aerogel from sodium carboxymethyl

cellulose/collagen composite aerogel for dye adsorption and electrochemical supercapacitor. *Int. J. Biol. Macromol.* **2018**, 115, 185-193.

S33 Akar, E.; Altinisik, A.; Seki, Y. Using of activated carbon produced from spent tea leaves for the removal of malachite green from aqueous solution. *Ecol. Eng.* **2013**, 52, 19-27.

S34 Hameed, B.H.; El-Khaiary, M.I. Equilibrium, kinetics and mechanism of malachite green adsorption on activated carbon prepared from bamboo by K_2CO_3 activation and subsequent gasification with CO_2. *J. Hazard. Mater.* **2008**, 157, 344-351.

S35 Zhang, J.; Liu, M.; Liu, Z.; Yang, T.; He, Q.; Yang, K.; Wang, H. Studies of malachite green adsorption on covalently functionalized Fe_3O_4@SiO_2–graphene oxides core–shell magnetic microspheres. *J. Sol-Gel Sci. Technol.* **2017**, 82, 424–431.

S36 Hosseinzadeh, H.; Ramin, S. Fabrication of starch-graft-poly (acrylamide)/graphene oxide/hydroxyapatite nanocomposite hydrogel adsorbent for removal of malachite green dye from aqueous solution. *Int. J. Biol. Macromol.* **2018**, 106, 101-115.

S37 Ghasemi, M.; Mashhadi, S.; Asif, M.; Tyagi, I.; Agarwal, S.; Gupta, V.K. Microwave-assisted synthesis of tetraethylenepentamine functionalized activated carbon with high adsorption capacity for Malachite green dye. *J. Mol. Liq.* **2016**, 213, 317-325.

S38 Sarkar, A.K.; Pal, A.; Ghorai, S.; Mandre, N.R.; Pal, S. Efficient removal of malachite green dye using biodegradable graft copolymer derived from amylopectin and poly(acrylic acid). *Carbohyd. Polym.* **2014**, 111, 108-115.

S39 Zhou, Y.; Min, Y.; Qiao, H.; Huang, Q.; Wang, E.; Ma, T. Improved removal of malachite green from aqueous solution using chemically modified cellulose by anhydride. *Int. J. Biol. Macromol.* **2015**, 74, 271–277.

S40 Sykam, N.; Madhavi, V.; Rao, G.M. Rapid and efficient green reduction of graphene oxide for outstanding supercapacitors and dye adsorption applications. *J. Environ. Chem. Eng.* **2018**, 6, 3223-3232.

S41 Gupta, K.; Khatri, O.P. Reduced graphene oxide as an effective adsorbent for removal of malachite green dye: Plausible adsorption pathways. *J. Colloid Interf. Sci.* **2017**, 501, 11-21.

S42 Yakout, A.A. Shaker, M.A. Dodecyl sulphate functionalized magnetic graphene oxide nanosorbent for the investigation of fast and efficient removal of aqueous malachite green. *J. Taiwan Inst. Chem. E.* **2016**, 63, 81-88.

S43 Lin, F.; You, Y.; Yang, X.; Jiang, X.; Lu, Q.; Wang, T.; Huang, B.; Lu, B. Microwave-assisted facile synthesis of TEMPO-oxidized cellulose beads with high adsorption capacity for organic dyes. *Cellulose* **2017**, 24, 5025-5040.

S44 Baghdadi, M.; Soltani, B.A.; Nourani, M. Malachite green removal from aqueous solutions using fibrous cellulose sulfate prepared from medical cotton waste: Comprehensive batch and column studies. *J. Ind. Eng. Chem.* **2017**, 55, 128–139.

S45 Chen, Y.; Lin, Y.C.; Ho, S.H.; Zhou, Y.; Ren, N. Highly efficient adsorption of dyes by biochar derived from pigments-extracted macroalgae pyrolyzed at different temperature. *Bioresource Technol.* **2018**, 259, 104–110.
